# Paramacular Acute Middle Maculopathy Associated with Glyceryl Trinitrate

**DOI:** 10.1155/2021/8215706

**Published:** 2021-09-18

**Authors:** Tryfon Rotsos, Ioannis Giachos, Konstantinos Tyrlis, Chrysanthos Symeonidis, Ekaterini Mani, Ilias Georgalas

**Affiliations:** ^1^1st Department of Ophthalmology, National and Kapodistrian University of Athens, Athens, Greece; ^2^2nd Department of Ophthalmology, Aristotle University of Thessaloniki, Thessaloniki, Greece

## Abstract

An unusual case of nitroglycerin-induced Paracentral Acute Middle Maculopathy (PAMM) is presented. A 50-year-old patient with sudden vision loss and scotoma was followed up with swept-source optical coherence tomography (SS-OCT), optical coherence tomography-angiography (OCT-A), and fluorescein angiography (FA). An anal fissure treated with glyceryl trinitrate (GTN) 0.2% ointment with headache and dizziness after application was reported. Fundoscopy OS revealed mild retinal venous dilatation and tortuosity with scattered blot hemorrhages and subtle, parafoveal, whitish lesions in the outer retina. SS-OCT revealed diffuse, hyperreflective lesions in the inner plexiform (IPL), inner nuclear (INL), and outer plexiform layers (OPL). OCT-A revealed focal dropout in the deep capillary plexus. FA showed masking due to blot hemorrhages and early punctuate leakage in the inner retina. This entity was identified as nitroglycerin-induced PAMM. Over the following 8 months, after discontinuation of the ointment application, the patient was symptom-free with stable visual acuity. OCT revealed INL/OPL thinning and confirmed complete lesion resolution. This first report of retinal vascular abnormalities due to nitrite ointment provides an insight into an unknown side effect of nitroglycerin ointment use. A dose-dependent correlation between GTN application and retinal vascular abnormalities remains to be confirmed.

## 1. Introduction

Glyceryl trinitrate (GTN) is a nitric oxide-releasing drug which is mainly used in the management of angina pectoris, acute myocardial infarction, severe hypertension, and acute coronary artery spasms by dilating the venous system and decreasing ventricular preload. GTN is a prodrug which after denitration produces the active metabolite nitric oxide (NO). Organic nitrates that undergo these two steps within the body are called nitrovasodilators. GTN has also applications in the treatment of benign anal diseases such as anal ulcer and hemorrhoids [1]. An abnormal pressure response of the internal anal sphincter (IAS) plays a role in the pain sensation that characterizes these lesions. In the setting of anal fissure, chemical sphincterotomy leads to reduced anal canal pressure, relieves pain, and promotes localized healing. The IAS is innervated by nitric oxide-releasing nerves, and the release of NO triggers IAS to relaxation [2]. Administration of exogenous NO, via glyceryl trinitrate, has a similar effect [3].

The most common side effect of topical GTN is headache [1]. This is indicative of systemic absorption of the drug and its potential for clinical manifestations in other organs. Specifically, in the eye, nitroglycerin has the ability to cross the blood-ocular barrier and have a vasodilatory effect to the retinal vessels [4]. In contrast to retinal arteries, retinal veins and capillaries lack any elastic or smooth muscle tissue [5]. They are, however, covered with pericytes that express a contractile filamentous component called alpha-smooth muscle actin (*α*SMA). This component acts as a major indicator of vascular smooth muscle cells and is the reason why retinal capillaries are affected by vasoregulators like GTN [6].

The case of a patient with possible nitroglycerin-induced Paracentral Acute Middle Maculopathy (PAMM) after treatment with nitroglycerin ointment is presented [7]. To our best knowledge, this is the first report of a patient presenting with retinal and vascular abnormalities due to nitrite ointment.

## 2. Case Report

A 50-year-old Caucasian male presented to the emergency department, complaining of sudden vision loss and the presence of scotoma in his left eye (OS) with no ocular pain or discomfort. His past ophthalmic history was unremarkable. The patient had a history of painful anal fissure under treatment with GTN 0.2% ointment three times daily for the previous 2 months. After almost every application of the ointment, he was symptomatic with headache and dizziness that were partially relieved by common analgesics. He also reported that during the previous 15 days, pain from the fissure was significantly stronger, especially after bowel movement, which caused him to apply the ointment more often (up to 6 times daily). During the last 3 days, he reported episodes of transient postural hypotension. He was under no other medications and reported only occasional consumption of caffeine. He had no history of smoking, excessive drinking, or substance abuse.

**Figure 1:**
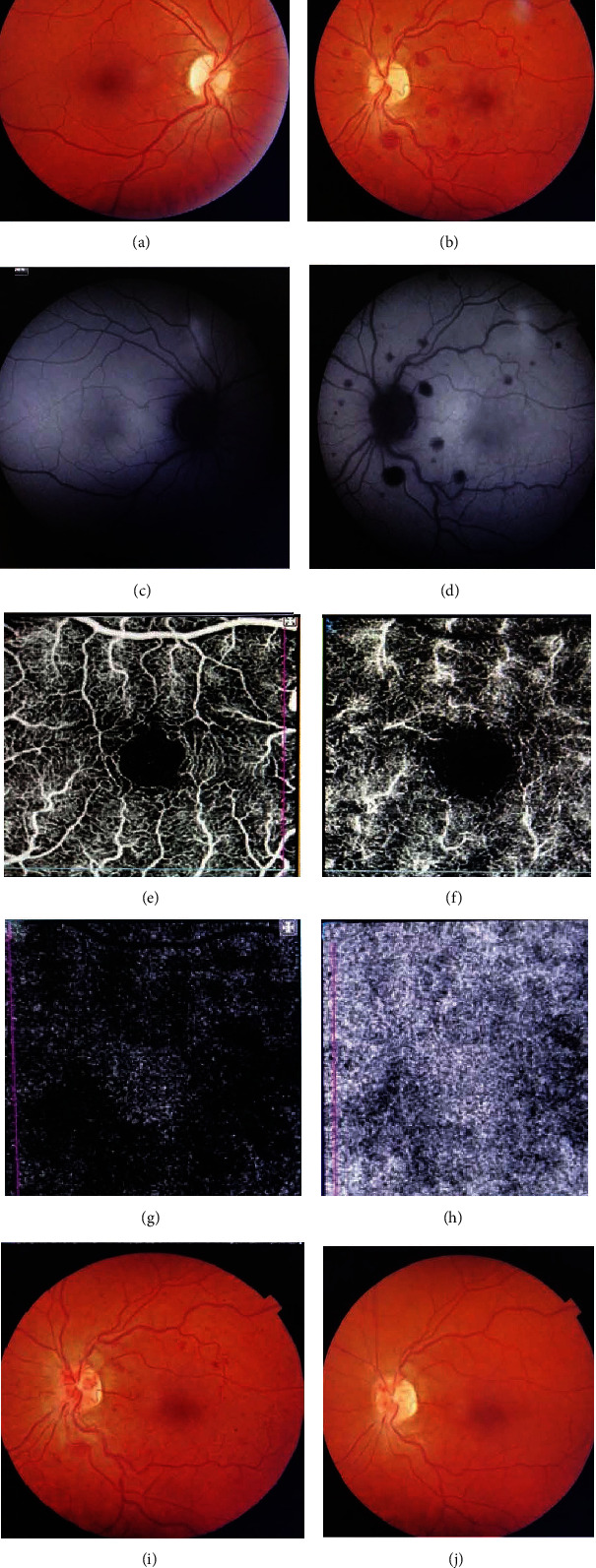
Fundus photo OD with no evidence of pathology (a). Fundus photo OS with mild retinal venous dilatation and tortuosity with scattered blot hemorrhages (b). Fundus autofluorescence does not reveal hyperfluorescent lesions or RPE changes (c, d) apart from the masking effect from blot hemorrhages OS (d). Optical coherence tomography-angiography demonstrates a focal, small area of capillary dropout in the deep capillary plexus (f). Superficial capillary plexus, outer retina, and choriocapillaris appear normal (e, g, h). Fundus photo OS, 1 month after presentation with fewer blot hemorrhages (i). Fundus photo after 6 months of OS shows complete resolution of the hemorrhages (j).

Best-corrected visual acuity (BCVA) was 85 ETDRS letters OD and 75 ETDRS letters OS. Intraocular pressure was within normal limits. RAPD was negative in both eyes, and Ishihara plates showed no apparent dyschromatopsia. Anterior chamber examination of both eyes was unremarkable. There were no significant fundoscopic findings OD. Fundoscopy OS revealed mild retinal venous dilatation and tortuosity with scattered blot hemorrhages (Figures 1(a) and 1(b)). In the outer retina, subtle, parafoveal, whitish lesions were noted. Extensive multimodal imaging which included OCT, fundus autofluorescence (FAF), optical coherence tomography-angiography (OCT-A), and fluorescein angiography (FA) was performed (Figures 1(c)–1(h)).

**Figure 2:**
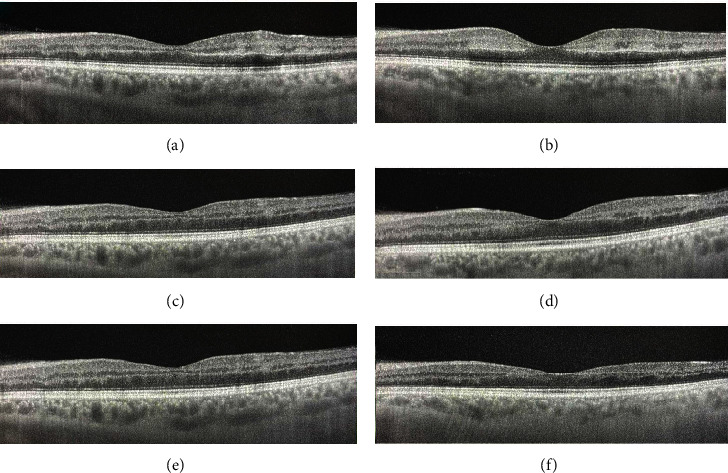
Swept-source- (SS-) OCT imaging of the left eye. Horizontal scans are presented on the left column and vertical ones on the right one (lines 1 and 6 on DRI OCT Triton, SS-OCT, respectively). OCT scans on presentation demonstrated diffuse, hyperreflective lesions at the level of the inner plexiform layer, inner nuclear layer, and outer plexiform layer (a, b). Partial resolution (one month later) (c, d) and complete resolution (six months later) (e, f) of the lesions are observed along with thinning of the inner retina.

**Figure 3:**
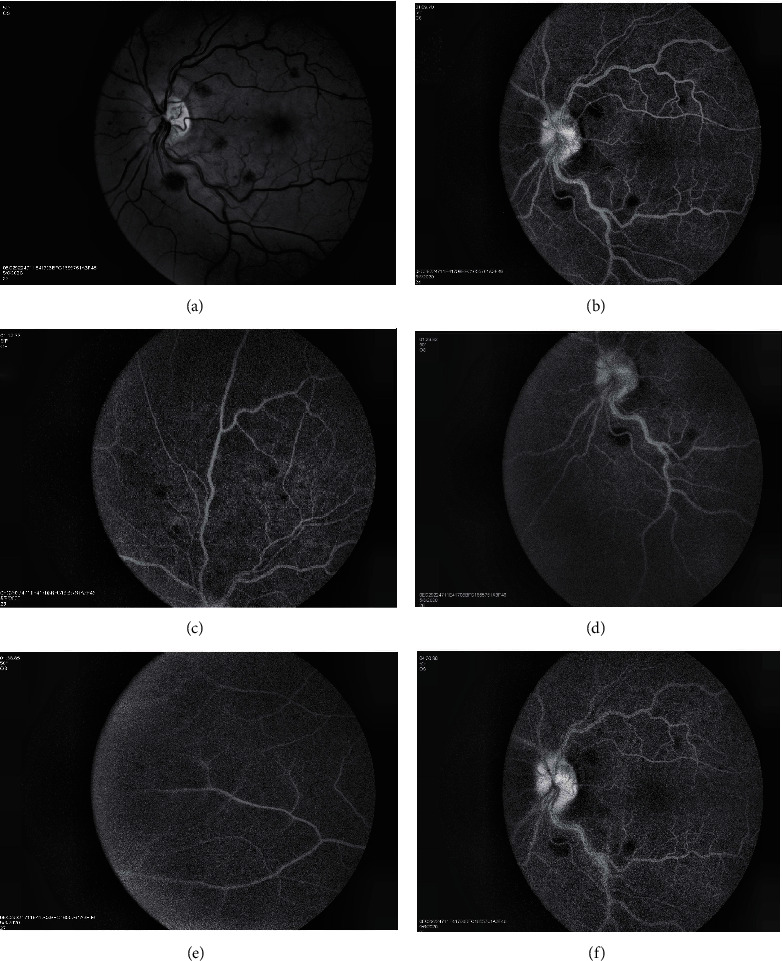
Fluorescein angiography (FA) images OS shows a masking effect due to blot hemorrhages and early punctuate leakage in the upper retina: (b) 1.09 min, (c) 1.19 min, (d) 1.29 min, (e) 1.38 min, and (f) 4.30 min.

Swept-source-optical coherence tomography (DRI OCT Triton, SS-OCT) demonstrated diffuse, hyperreflective lesions at the level of the inner plexiform layer (IPL), inner nuclear layer (INL), and outer plexiform layer (OPL) compatible with Paracentral Acute Middle Maculopathy (PAMM, Figures 2(a) and 2(b)). Fundus autofluorescence did not reveal any hyper- or hypofluorescent lesions of the Retinal Pigment Epithelium (RPE). Optical coherence tomography-angiography (DRI OCT Triton, SS-OCT) revealed a focal, small area of capillary dropout in the deep capillary plexus. Superficial capillary plexus, outer retina, and choriocapillaris appeared normal. No other vascular abnormalities were apparent. Fluorescein angiography revealed normal arteriovenous transit time, masking effect due to blot hemorrhages, and early punctuate leakage in the inner retina (Figure 3).

An extensive haematological workup which included complete blood count, fasting blood sugar and hemoglobin A1c, ESR, CRP, LDL, HDL, plasma homocysteine level, rheumatoid factor, ANA, ANCA, SACE, FTA-ABS, VDRL, antiphospholipid antibodies, protein C and S levels, factor V Leiden, cryoglobulins and a thrombophilia screen was performed. Moreover, plasma protein electrophoresis, chest X-ray (for the exclusion of sarcoidosis, tuberculosis, and left ventricular hypertrophy), and a magnetic resonance angiography (for any vascular stenosis) with contrast dye were also performed. The patient was also referred to cardiology for a full assessment with electrocardiogram, carotid Doppler imaging, cardiac echocardiogram, and rhythm and blood pressure Holter monitoring. There was no cardiological or vascular disease diagnosed. Haematological workup, X-ray, and MRA revealed no pathological findings.

The patient was advised to immediately discontinue any application of the drug. During the following month, the patient did not experience any of his previous symptoms and his visual acuity OS was 80 ETDRS letters with no RAPD. Fundoscopy revealed partial resolution of blot hemorrhages. The macula appeared normal. OCT revealed retinal disruption and thinning at the level of INL and OPL and partial resolution of the hyperreflective lesions (Figure 2(c)). OCT-A remained unaltered (Figure 1(i)).

Six months later, the patient was free of symptoms. Visual acuity OS was 80 ETDRS letters. Fundoscopy revealed complete resolution of the hemorrhages and the whitish lesions (Figure 1(j)). OCT showed INL and OPL thinning and complete resolution of the hyperreflective lesions (Figure 2(d)).

The patient mentioned that 8 months after the initial diagnosis, he had an exacerbation of symptoms regarding his anal fissure. He applied the ointment twice during a day for 4 days, which led to the remission of headaches and transient reduction of his visual acuity. He discontinued the drug application.

## 3. Discussion

Based on the clinical findings, multimodal imaging approach, laboratory results, and medical history of the patient, this disorder was identified as nitroglycerin-induced PAMM. Differential diagnosis included central retinal vein occlusion, branch retinal vein occlusion, ocular ischemic syndrome, retinal angiitis, diabetic retinopathy, and hypertensive retinopathy. Diabetic retinopathy and hypertensive retinopathy were excluded on the basis that the patient did not suffer from diabetes mellitus or hypertension and on the fact that the right eye remained asymptomatic and with no clinical signs. Central or branch retinal vein occlusion was excluded because of the atypical fundoscopic and fluorescein angiography findings, the presence of PAMM on presentation, the correlation of visual symptoms with the application of the ointment and improvement of symptoms, and findings after discontinuation of the drug. Ocular ischemic syndrome was excluded since the carotid Doppler ultrasound imaging and MRA were normal. A wide array of imaging and laboratory examinations were performed which failed to identify any obvious ocular disease. Valsalva retinopathy was excluded on the basis that the patient reported no episodes of constipation, only pain during defecation.

To our knowledge, this is the first reported case of nitroglycerin-related PAMM. It is conceivable that the disorder is a result of two possible mechanisms of action, a direct and an indirect one.

The direct mechanism of action involves the nitrate effect on the retinal vessels. As previously stated, nitroglycerin has the ability to cross the blood-ocular barrier and have a vasodilatory effect to the retinal vessels [4], affecting retinal capillaries and veins despite the lack of any elastic or smooth muscle tissue [6]. This effect can explain the retinal vein dilation. It is conceivable that the vasodilatory effect of nitroglycerin in this patient was so potent that it also facilitated dilation of the retinal capillaries of the superior and inferior temporal branches of the central retinal artery to such a degree, causing hypoperfusion. This hypoperfusion led in turn to ischemia at the level of IPL, INL, and OPL, which resulted in PAMM. The fact that the patient had significant symptoms after almost every application of the drug further supports this hypothesis.

The indirect mechanism involves the nitrate effect on the circulatory system. At high doses, nitrates are commonly used for the treatment of acute heart failure by venous and arterial dilation. Nitrates lower left ventricular filling pressures and reduce systemic resistance. This provides not only preload but also afterload reduction and resolution of symptoms. However, there have been reports of nitroglycerin-induced hypotension and bradycardia [8]. This patient reported episodes of transient postural hypotension. This hypotension may lead to hypoperfusion of the retina at the level of the retinal capillaries leading in turn to macula ischemia and PAMM.

There has been a report of PAMM induced by a different vasodilating drug (PDE5 inhibitor) which further supports our hypothesis [9].

A reasonable counter argument against our diagnosis would be the unilateral presentation of the disease. One could argue that since there is systemic absorption of the drug, both eyes should be roughly equally affected. We believe that since the patient applied the ointment for a relatively short period of time (i.e., two months) and used it excessively for only 15 days; it is justifiable to assume that there was not enough time for the changes to manifest in both eyes. If this type of usage continued, it is possible that both eyes would have similar manifestations.

This is the first report of retinal and vascular abnormalities due to nitrite ointment. Since this type of ointment is widely used for the treatment of anal fissures and hemorrhoids, this case report should raise concerns regarding optimal use of this drug. Patients who have underlying cardiovascular risk factors and/or retinal diseases should be more closely monitored and be advised to seek immediate ophthalmic evaluation, should any visual disturbances occur. Patients who are otherwise healthy should also be advised of potential (albeit rare) visual disturbances especially after overuse. This case report may provide a new insight into an unknown side effect of nitroglycerin ointment. Further studies are needed to confirm a potential correlation between GTN application and vascular abnormalities of the retina.

## Data Availability

Relevant data is available on request. Please contact Dr. Chrysanthos Symeonidis (chrys2209@gmail.com).
